# B-type natriuretic peptide versus amino terminal pro-B type natriuretic peptide: selecting the optimal heart failure marker in patients with impaired kidney function

**DOI:** 10.1186/1471-2369-14-117

**Published:** 2013-05-31

**Authors:** Lena Jafri, Waqar Kashif, Javed Tai, Imran Siddiqui, Iqbal Azam, Hira Shahzad, Farooq Ghani

**Affiliations:** 1 Department of Pathology & Microbiology, Aga Khan University, Stadium Road, P.O. Box 3500, Karachi 74800, Pakistan; 2 Department of Medicine, Aga Khan University, Stadium Road, P.O. Box 3500, Karachi 74800, Pakistan; 3 Department of Community Health Services, Aga Khan University, Stadium Road, P.O. Box 3500, Karachi 74800, Pakistan; 4 Medical College Aga Khan University, Stadium Road, P.O. Box 3500, Karachi 74800, Pakistan

**Keywords:** B-Type natriuretic peptide, Heart failure, NT-proBNP, Kidney

## Abstract

**Background:**

The effect of impaired kidney function on B-type natriuretic peptide (BNP) and N-terminal proBNP (NT-proBNP) is vague. This study was performed to examine the effect of kidney dysfunction on the afore-mentioned markers and determine appropriate cutoffs for systolic heart failure (SHF).

**Methods:**

In this cross sectional study adults with estimated glomerular filtration rate (eGFR) <60 ml/min for ≥3 months were identified in consulting clinics from June 2009 to March 2010. SHF was defined as documented by a cardiologist with ejection fraction of < 40% and assessed by New York Heart Association classification (NYHA). Plasma was assayed for creatinine (Cr), BNP and NT-proBNP.

**Results:**

A total of 190 subjects were enrolled in the study, 95 with and 95 without SHF. The mean age of patients was 58 (±15) years, 67.4% being males. Mean BNP levels showed a 2.5 fold and 1.5 fold increase from chronic kidney disease (CKD) stage 3 to stage 5 in patients with and without SHF respectively. NT-proBNP levels in non-heart failure group were 3 fold higher in CKD stage 5 compared to stage 3. Mean NT-proBNP levels were 4 fold higher in CKD stage 5 compared to stage 3 in patients with SHF. Optimal BNP and NT-proBNP cutoffs of SHF diagnosis for the entire CKD group were 300 pg/ml and 4502 pg/ml respectively.

**Conclusion:**

BNP and NT-proBNP were elevated in kidney dysfunction even in the absence of SHF; however the magnitude of increase in NT-proBNP was greater than that of BNP. BNP and NT-proBNP can be useful in diagnosing SHF, nonetheless, by using higher cutoffs stratified according to kidney dysfunction. NT-proBNP appears to predict heart failure better than BNP.

## Background

Literature from the United States, Australia and China report the prevalence of chronic kidney disease (CKD) as ranging from 11–13.1% [1-4]. Community based studies in Pakistan reveal a high burden of CKD ranging from 15 to 20% in subjects older than 40 years of age [5]. The exact prevalence of CKD in Pakistan is still unknown due to lack of documentation and funds, but it is expected to be high, with respect to the observation of the epidemic of diabetes and hypertension in this part of the world [6-8]. The prevalence of heart failure increases as glomerular filtration rate (GFR) declines and as many as 35% of patients reaching end stage renal disease already have clinical evidence of heart failure [9]. To prevent the occurrence of heart failure a reliable marker for observing cardiac overload in such patients is needed. The B-type natriuretic peptide (BNP) and N- terminal pro-hormone B-type natriuretic peptide (NT-proBNP) are established heart failure markers but concomitant presence of CKD changes their interpretation in significant manner [10-13].

The source of BNP and NT-proBNP are mainly left ventricular myocytes. Distention of cardiac ventricle is considered the main stimulus for release of proBNP_1-108._ This pro-hormone is released into the circulation and is proteolytically cleaved into the biologically active BNP_1-32_ and the inactive NT-proBNP_1-76_. The understanding of the cleavage of proBNP in circulation is most likely by the pro-protein convertases corin and furin [14,15]. The processing of proBNP is indeed complex, with significant release of unprocessed proBNP, particularly in heart failure. Several recent studies have demonstrated that there are only small amounts of intact BNP in blood, and the major circulating forms of BNP are degradation products. These degradation products and intact proBNP are detected by BNP assays to a varying extent [16]. The synthesis and release of BNP is controlled at the level of gene expression which is predominantly controlled by ventricular hypertrophy, inflammation or stretch [17]. The clearance mechanism of BNP is through the endocytosis followed by lysosomal degradation, and through the degradation by the nonspecific membrane-bound enzyme neutral endopeptidase but NT-proBNP is mainly cleared via the kidneys [18-20]. Both are released in a 1:1 ratio but levels of NT-proBNP are higher than that of BNP because of half-life of 15–20 minutes whereas the half-life of NT-proBNP has been estimated to be longer (1–2 hours) [21,22].

We presume that with declining kidney function. NT-proBNP would be affected more as compared to BNP. The ideal natriuretic peptide to diagnose heart failure in CKD remains undecided. This study was conducted to evaluate the effects of compromised kidney function on natriuretic peptides (BNP or NT-proBNP) and to determine optimal cutoffs predictable of systolic heart failure (SHF).

## Methods

### Study population and procedure

A cross-sectional study was conducted in the Section of Chemical Pathology, Department of Pathology and Microbiology in collaboration with the nephrology and cardiac units of Aga Khan University, Karachi Pakistan. It was conducted over a period of 10 months from June 2009 to March 2010. The Aga Khan University’s Ethical Review Committee approved all investigational procedures involved in the study (reference number: 1054-Path-ERC-08). Recruitment of consecutive adult ambulatory subjects with impaired kidney function was carried out via non-probability quota sampling from the clinics. After taking informed consent a proforma was filled including patient demographics, clinical history, smoking history, history of alcohol intake and drug history. The patient’s body weight and height were recorded in order to determine the body mass index (BMI).

Impaired kidney function was taken as estimated glomerular filtration rate (eGFR) of ≤ 60 ml/min. The Cockcroft Gault equation was used to estimate the GFR of the study subjects from plasma creatinine (Cr), age and body weight of the subjects [23]. The subjects stratification was carried out into CKD stage 3 (eGFR 30–60 ml/minute), stage 4 (eGFR 15–29 ml/minute), and stage 5 (eGFR <15 ml/minute) [24]. Patients were classified as having SHF as per the interview data and physical examination using New York Heart Association (NYHA) and then reconfirmed from past echocardiography report (ejection fraction <40%) or SHF documented on the file by a cardiologist [25]. Patients on dialysis therapy and obese patients with body mass index >30 kg/m^2^ were not included in this study. Both endogenous and exogenous hormones have shown to induce elevated levels of BNP/NT-proBNP in circulation therefore pregnant females, those on hormonal therapy or taking contraceptives were also not included in this study group [26,27]. Six milliliter of blood sample was taken from every participating patient into vacutainers containing ethylene diamine tetra acetic acid (EDTA) for analysis. Plasma was obtained after centrifugation of samples for analyzing BNP, NT-proBNP and creatinine.

### Biochemical measurements

Plasma samples were collected after informed consent and centrifuged at 3000 g for 10 minutes, aliquoted, frozen and maintained at −80°C. All samples were run in batches for plasma BNP, NT-pro BNP and Cr. The BNP levels were measured by automated electrochemiluminescence immunoassay (ECLIA) on Axym analyzer (Abbott diagnostic). The NT-proBNP levels were measured by automated ECLIA on Elecsys analyzer (Roche diagnostic). Plasma Cr levels were assayed with the rate-Jaffe reaction (with a calibrator traceable to the isotope dilution mass spectrometry reference method) on Synchron analyzer (Beckman Coulter). To ensure quality, normal and abnormal controls were run with every batch of BNP, NT-proBNP and Cr analysis.

### Statistical analysis

Data analysis was performed using SPSS version 19. After checking for normality using the Kolomogorov-Smirnove test, BNP and NT-proBNP values were log transformed. Mean and ± standard deviation (SD) or median with 25^th^ to 95^th^ percentile for continuous variables were computed. Dichotomous variables were expressed in percentages. The differences between groups were tested using *t*-test, one way analysis of variance (ANOVA) or Chi square test, as applicable. Post-hoc comparisons of log BNP/NT-proBNP in different groups were carried out using Bonferroni method. Variables used in univariate analysis with *p*-value of < 0.2 were then entered in a multiple regression model using ENTER method. Results were reported as standardized ß-coefficient. Receiver operating characteristic (ROC) curves were constructed and areas under the curve (AUC) calculated for BNP and NT-proBNP to detect SHF. Compromising on sensitivity in order to improve the specificity the uppermost corner on the left side of the ROC curve was not used for calculating cutoffs. A *p*-value of < 0.05 was treated as significant in all above statistical analysis.

## Results

### Study population

Table 1 depicts the characteristics, medical history and biochemical results of one hundred and ninety study subjects enrolled in this study. The EF of all the study subjects was available. The mean age of the study population was 58 ± 15 years and the majority was males (67.4%). The median duration of impaired kidney function in subjects with impaired kidney function with and without SHF was 18 months (Interquartile Range = 3 – 160 months) and 24 months (Interquartile Range = 6 – 190 months) respectively (*p*-value > 0.05). Statistically non-significant difference existed between mean log BNP/NT-proBNP amongst males and females in those with and without SHF (*p*-value >0.05).

**Table 1:** Clinical characteristics and biochemical data of patients with impaired kidney function stratified into two groups based on cardiac function

**Characteristics of total subjects**				**p value**
	**Mean (±SD), n (%)**			
	**Overall**	**Without SHF**	**With SHF**	
	**n = 190**	**n = 95**	**n = 95**	
**Age (years)**	58 ± 15	53 ± 15	63 ± 14	0.00
**Male gender (%)**	128 (67.4)	59 (62.1)	69 (72.6)	0.12
**BMI (kg/m**^**2**^**)**	23 ± 3.1	24.1 ± 3.2	21.9 ± 2.5	0.00
**Hypertension (%)**	141 (74.6)	71 (74.7)	70 (73.7)	0.77
**Anemia (%)**	129 (67.9)	64 (67.4)	65 (68.4)	0.87
**Diabetes (%)**	108 (56.8)	49 (51.6)	59 (62.1)	0.14
**Dyslipidemia (%)**	69 (36.3)	33 (34.7)	36 (37.9)	0.65
**Ischemic heart disease (%)**	39 (20.5)	0	39 (41.1)	0.00
**Smoking history (%)**	6 (3.2)	1 (1.1)	5 (5.3)	0.09
**Cerebrovascular accident (%)**	4 (2.1)	1 (1.1)	3 (3.2)	0.31
**Malignancy (%)**	1 (0.5)	0	1 (1.1)	0.31
**Alcohol intake (%)**	1 (0.5)	1 (1.1)	0	0.31
**ACE inhibitors**	91 (47.9)	51 (53.7)	40 (42.1)	0.11
**Statins**	69 (36.3)	33 (34.7)	36 (37.9)	0.65
**Calcium channel blockers**	49 (25.8)	32 (33.7)	17 (17.9)	0.01
**Diuretics**	41 (21.6)	19 (20)	22 (23.2)	0.59
**Digoxin**	22 (11.6)	0	22 (23.2)	0.00
**ARB**	20 (10.5)	8 (8.4)	12 (12.6)	0.34
**Beta blocker**	13 (6.8)	12 (12.6)	1 (1.1)	0.00
**Stage 3 CKD (%)**	77 (40.5)	41 (43.2)	36 (37.9)	0.75
**Stage 4 CKD (%)**	77 (40.5)	37 (38.9)	40 (42.1)	0.75
**Stage 5 CKD (%)**	36 (18.9)	17 (17.9)	19 (20)	0.49
**Ejection fraction (%)**	42.9 ± 6.8	59.3 ± 3.5	26.6 ±10.2	0.00
**eGFR (ml/min)**	27.7 ± 14	29.1 ± 15.1	26.2 ± 12.9	0.15
**Cr (mg/dl)**	3.2 ± 2.2	3.3 ± 2.4	3.0 ± 1.8	0.34
**Log BNP (pg/ml)**	2.2 ± 0.5	2.0 ± 0.4	2.4 ± 0.6	0.00
**Log NT-proBNP (pg/ml)**	3.4 ± 0.6	2.9 ± 0.6	3.9 ± 0.6*	0.00

Abbreviations: *SHF* systolic heart failure, *SD* standard deviation, *BMI* body mass index, *ACE* angiotensin converting enzyme, *ARB* angiotensin receptor blocker, *CKD* chronic kidney disease, *eGFR* estimated glomerular filtration rate, *Cr* creatinine, *BNP* b type natriuretic peptide, *NT-proBNP* Amino terminal B-type natriuretic peptide.

### Multivariate predictors of natriuretic peptides

The following predictor variables were used in univariate analysis taking log BNP/log NT-proBNP as the dependent variables: age, gender, BMI, eGFR, ejection fraction, diabetes, hypertension, ischemic heart disease, cerebrovascular accident, dyslipidemia, anemia, smoking history, calcium channel blockers, beta-blockers, statins, digoxin, angiotensin converting enzyme inhibitor, angiotensin receptor blocker, diuretics and duration of kidney disease. Table 2 shows the final multivariate regression model for both BNP and NT-proBNP based on total number of subjects. Significant predictors of log NT-proBNP were found to be age, BMI, SHF, NYHA stage, and eGFR. Concentration of NT-proBNP increased progressively with declining kidney function estimated as eGFR. The log BNP and log NT-proBNP showed inverse correlation with EF (r = −0.3 and −0.6 respectively). Both BNP and NT-proBNP significantly correlated with NYHA functional classification.

**Table 2:** Predictors of log NT-proBNP in multivariate regression analysis

**Variables**	**Predictors of log BNP**			**Predictors of log NT-proBNP**		
	**B standardized coefficient**	**Confidence Interval**	**p value**	**B standardized coefficient**	**Confidence Interval**	**p value**
**Age**	0.055	−0.003 to 0.007	0.40	−0.108	−0.011 to 0.000	0.03
**Creatinine**	−0.030	−0.054 to 0.038	0.74	−0.079	−0.085 to 0.024	0.26
**Body Mass Index**	0.008	−0.014 to 0.031	0.46	0.104	0.003 to 0.053	0.02
**Duration of kidney disease**	0.095	0.000 to 0.004	0.12	−0.029	−0.003 to 0.002	0.53
**Heart Failure**	−0.134	−0.445 to 0.147	0.32	0.262	0.077 to 0.800	0.01
**NYHA stage**	0.075	−0.089 to 0.165	0.55	0.634	0.368 to 0.617	0.00
**Ejection fraction**	0.045	−0.008 to 0.010	0.76	0.138	−0.004 to 0.017	0.22
**eGFR**	0.070	−0.005 to 0.011	0.48	−0.468	−0.036 to −0.019	0.00
**Log BNP/NT-proBNP**	0.729	0.358 to 0.615	0.00	0.468	0.341 to 0.595	0.00

Abbreviations: *BNP* b-type natriuretic peptide, *NT-proBNP* amino terminal B-type natriuretic peptide, *NYHA* New York Heart Association, *eGFR* estimated glomerular filtration rate, *ARB* angiotensin receptor blocker.

p-value <0.05 statistically significant.

### Comparison of natriuretic peptides in NYHA groups

According to NYHA classification 35.8% were in NHYA 1, 19.5% in NYHA 2, 30% in NYHA 3 and 14.7% belonged to NYHA 4. Median BNP in these four classes were 80.8 (42.6-163.2), 200 (118.7-331), 478 (144.1 - 742.7), and 409.4 (285.8-720.3) pg/ml respectively. Post-hoc test was applied for multiple comparisons, using Bonferroni alpha (0.05), which revealed significant differences in log BNP between NYHA Class I, II and III whereas differences were non- significant between NYHA Class III and IV. Median log NT-proBNP in NYHA classes were 535.1 (191.9- 876.), 4192 (1505.8-7105), 13831 (4640.5-31230.5) and 22520 (9693.2-35000) pg/ml respectively. There was a significant mean difference in ANOVA results between log NT-proBNP levels amongst all the four classes of NYHA classification (*p*-value <0.05). A progressive decline in eGFR with a rise in NYHA class was noted (r = − 0.2, *p*-value <0.05).

### Renal dysfunction and natriuretic peptides

**Figure 1:**
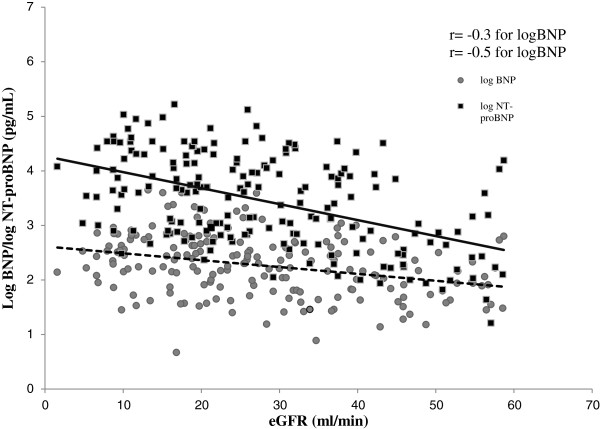
**Scatter plot of log transformed plasma B-type natriuretic peptide (BNP) and amino terminal B-type natriuretic peptide (NT-proBNP) in relation to estimated glomerular filtration rate (eGFR).** Lines of best linear fit are drawn.

Both natriuretic peptides showed rising trend as eGFR declined as depicted in Figure 1. Median BNP values in CKD 3, 4 and 5 were 106.3 (43.1 - 281.6), 266.6 (108.9 - 589.3) and 328.9 (142.1 - 650.6) pg/ml respectively. Median NT-proBNP values in CKD 3, 4 and 5 were 799 (227 – 5230), 5004 (905.5 - 14776.5), and 11215.5 (3532.5 – 35000) pg/ml respectively.

**Figure 2:**
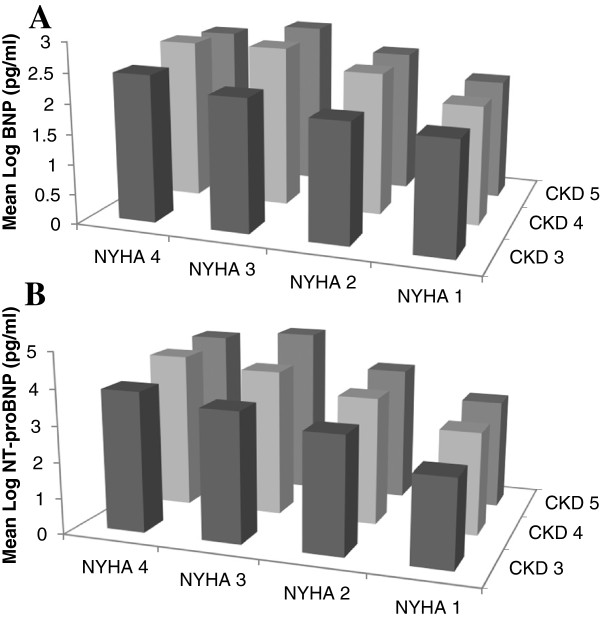
**Impact of chronic kidney disease and New York Heart Association Classification on natriuretic peptides. A**. Log BNP according to CKD stages and NYHA classification. **B**. Log NT-proBNP according to CKD stages and NYHA classification.

Plasma BNP and NT-proBNP levels increased stepwise as kidney function deteriorated and the levels were significantly higher as the SHF worsened (Figure 2A and B). Highest BNP levels were noted in CKD stage 5 along with NYHA class 3 and highest NT-proBNP concentration was noted in CKD stage 5 along with NYHA class 3.

A continuous relationship of both peptides was revealed by head to head comparison of log BNP and log NT-proBNP in the total group of patients. Log transformed BNP levels correlated with log transformed NT-proBNP levels (r = 0.43, *p*-value <0.05). The association of BNP with NT-proBNP was higher in those suffering from kidney dysfunction alone without SHF (r = 0.7, *p*-value <0.05) compared to those suffering from kidney dysfunction and SHF (r = 0.55, *p*-value <0.05). Non-significant correlation with each other was shown by both peptides in the group with SHF along with CKD 5.

Concentration of BNP and NT-proBNP were not interchangeable. The mean BNP levels showed a 1.5 fold increase from CKD stage 3 to CKD stage 5 in those without SHF. In patients with SHF, a 2.5 fold increase was observed in BNP levels as the CKD stage progressed from CKD stage 3 to 5. A marked rise in mean NT-proBNP levels was noted in non-heart failure group and levels were three fold higher in CKD stage 5 compared to CKD stage 3. Similarly mean NT-proBNP levels were four times higher in CKD stage 5 compared to CKD stage 3 in patients with SHF.

### Receiver operator curve (ROC) analysis and recommended cutoffs

**Figure 3:**
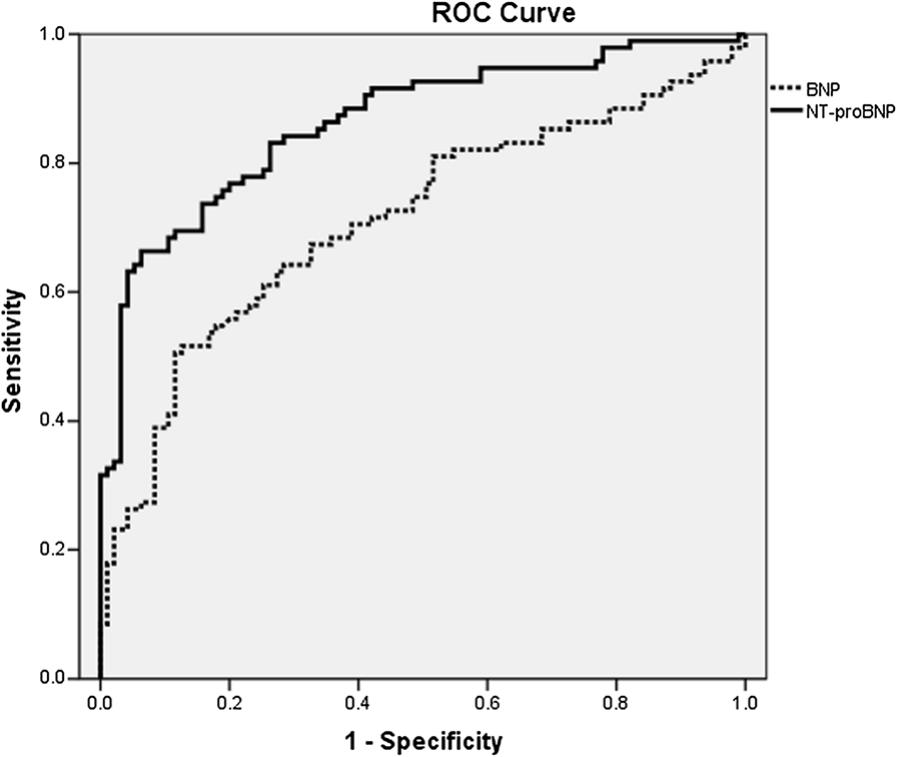
**Entire study group ROC curve of BNP and NT-proBNP for heart failure.** The area under the curve for BNP and NT-proBNP was 0.70 and 0.86 respectively.

**Figure 4:**
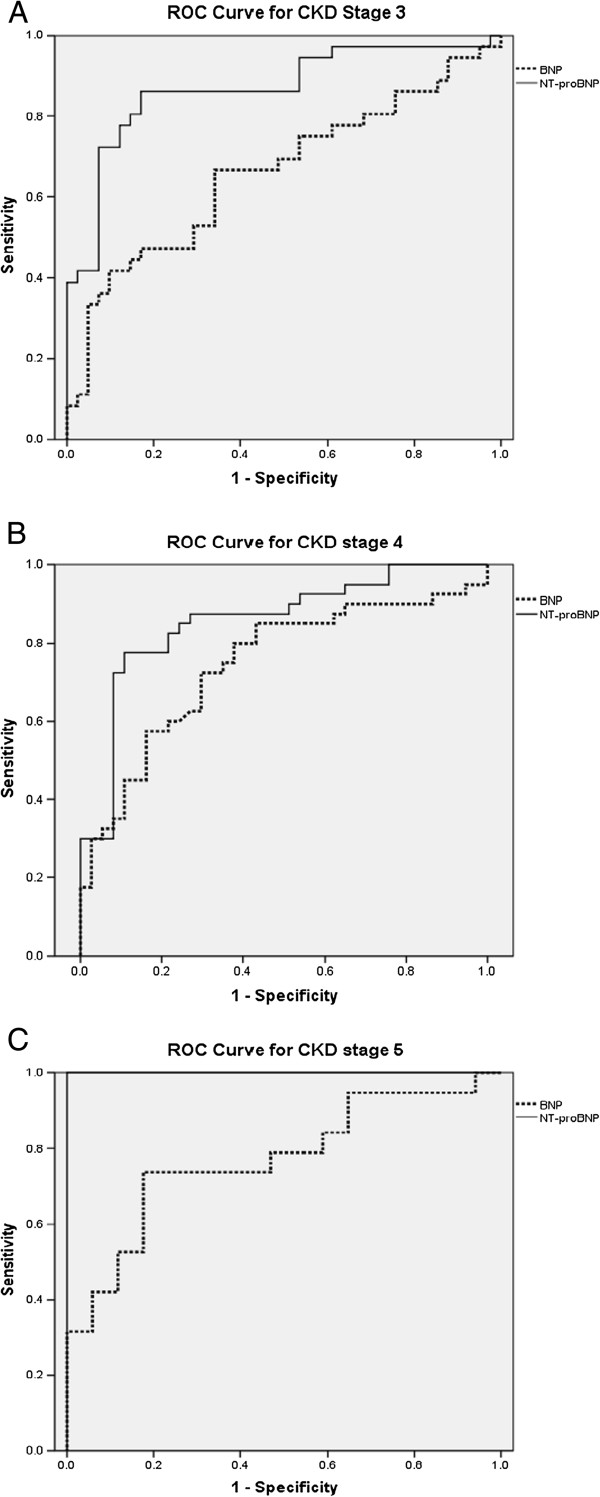
**Receiver-operating characteristic (ROC) curves of BNP and NT-proBNP according to chronic kidney disease (CKD) staging.** (**A**) For CKD stage 3 the area under the curve (AUC) for BNP and NT-proBNP was 0.66 and 0.88 respectively. (**B**) For CKD stage 4 the area under the curve (AUC) for BNP and NT-proBNP was 0.74 and 0.85 respectively. (**C**) For CKD stage 5 the area under the curve (AUC) for BNP and NT-proBNP was 0.77 and 1.00 respectively.

The assays for BNP and NT-proBNP remained sensitive and specific among patients with impaired kidney function as indicated by the AUC (Figure 3). On ROC analysis the AUC for BNP and NT-proBNP was 0.7 (95% CI 0.63 – 0.78, *p*-value <0.05) and 0.86 (95% CI 0.81 – 0.91, *p*-value <0.05) respectively (Figure 4). Figure 4A, B and C shows the ROC analysis of BNP and NT-proBNP according to CKD stratification. The optimal cutoffs of BNP and NT-proBNP for diagnosing SHF for the entire study group and also according to CKD staging are shown in Tables 3 and 4.

**Table 3:** Potential BNP and NT-proBNP cut off levels for the assessment of systolic heart failure for all CKD subjects

**Natriuretic peptide**	**Group**	**Optimal cut point (pg/ml)**	**Sensitivity**	**Specificity**	**Positive likelihood ratio**	**Negative likelihood ratio**
			**%**	**%**		
BNP	Overall	217	64.2	70	1.04	0.96
BNP	Overall	254	61	75	1.14	0.90
BNP	Overall	300	55	82.1	3.05	0.55
NT-proBNP	Overall	799	91.6	52.6	1.99	0.16
NT-proBNP	Overall	1536	84.2	71.6	4.13	0.30
NT-proBNP	Overall	4502	73.7	82.1	4.17	0.30

Abbreviations: *BNP* B-type natriuretic peptide, *NT-proBNP* amino terminal B-type natriuretic peptide, *AUC* area under the curve, *CKD* chronic kidney disease.

**Table 4:** Potential BNP and NT-proBNP cut off levels for the assessment of systolic heart failure according CKD staging

**Natriuretic peptide**	**Group**	**Optimal cut point (pg/ml)**	**Sensitivity**	**Specificity**
			**%**	**%**
**BNP**	Stage 3 CKD	146	60	65.9
**BNP**	Stage 4 CKD	309.4	60	78.4
**BNP**	Stage 5 CKD	491	60	88.2
**NT-proBNP**	Stage 3 CKD	1505.8	72.2	92.7
**NT-proBNP**	Stage 4 CKD	7767.5	70	91.9
**NT-proBNP**	Stage 5 CKD	11215.2	94.7	100

Abbreviations: *BNP* B-type natriuretic peptide, *NT-proBNP* amino terminal B-type natriuretic peptide, *AUC* area under the curve, *CKD* chronic kidney disease.

## Discussion

Multiple trials have reported that impaired kidney function augments the levels of these natriuretic peptides but the optimal cutoffs are not well known with various patients’ population. This is the first study from this part of the world and adds to the clinical utility of these cardiac markers in specific renal population. The study population represents typical outpatients with known kidney dysfunction, a highly relevant population group to explore the utility of BNP and NT-proBNP as heart failure markers. Current study addressed the utility of BNP and NT-proBNP as heart failure markers in kidney patients. The results of this study are in concordance with previous studies and revealed an inverse relationship between kidney function and BNP (r = − 0.3) and NT-proBNP (r = − 0.5) [12,28-31]. A head to head comparison between BNP and NT-proBNP revealed that NT-proBNP was much higher as compared to BNP in the SHF group and both values were not interchangeable. Using ROC analysis, the AUC for NT-proBNP was better than BNP in the entire study population, as well as in subgroups of CKD stages.

In this study group, plasma NT-proBNP levels were more affected by declining eGFR as compared to plasma BNP levels plus the optimal cut off for plasma NT-proBNP in the diagnosis of SHF was markedly influenced by the severity of kidney dysfunction. A great deal of controversy exists regarding the cut-off values of BNP and NT-proBNP levels in diagnosing SHF in patients with impaired kidney function. Generally the BNP and NT-proBNP values 100 pg/ml and 125 pg/ml are considered abnormal and suggestive of heart failure as per manufacturers’ product inserts. The strength of these markers is their capability to rule out the diagnosis of acute heart failure. In general, heart failure is unlikely at BNP values <100 pg/ml and is very likely at BNP values >500 pg/ml and, similarly, unlikely at NT-proBNP values < 300 pg/ml and very likely at NT-proBNP values >450 pg/ml [32-34]. McCullough et al. previously reported an analysis from Breathing Not Properly Multinational Study in which BNP levels were found to be related to kidney function in patients with and without heart failure [35]. McCullough et al. reported that it would be appropriate to apply diagnostic value of approximately 200 pg/ml for BNP in those with eGFR <60 ml/min/1.73 m^2^. Jae Wong Yang et al. stated that heart failure could be diagnosed in patients with kidney dysfunction using BNP cut-off 858.5 pg/ml with 77% sensitivity and 72% specificity [36]. Anwaruddin et al. (PRIDE Study) noted that heart failure could be diagnosed with 89% sensitivity and 72% specificity using NT-proBNP cut-off of 1200 pg/ml in patient with eGFR < 60 ml/min. It was reported that AUC for NT-proBNP in those with GFR > 60 ml/min was 0.95 \for detecting heart failure however, among those with GFR < 60 ml/min the NT-proBNP assay remained sensitive and specific with AUC of 0.88 [30]. AUC to detect heart failure tended to be greater for NT-proBNP than that for BNP in our study population.

Kidney dysfunction represents a variable that complicates the interpretation of BNP and NT-proBNP. As reported previously [29,30,37,38], findings of this study propose that higher BNP and NT-proBNP cut-off points would be needed to diagnose SHF in patients with impaired kidney function. We propose BNP and NT-proBNP cutoff levels of 300 pg/ml and 4502 pg/ml respectively; for diagnosing SHF in patients with eGFR <60 ml/min. Luchner et al. demonstrated lower cut-offs; 125 pg/ml for BNP and 350 pg/ml for NT-proBNP in post-myocardial patients with renal dysfunction. The assay used for NT-proBNP by them was similar to the one used in current study (Roche diagnostics) but the mean eGFR in the impaired kidney function group was 71 ± 12 ml/min and mostly included patients with mild renal dysfunction [31].

Our results show that NT-proBNP was more dependent on kidney function than BNP and but using a higher NT-proBNP cut-off had a better diagnostic accuracy than BNP. Given the high prevalence of left ventricular hypertrophy and left ventricular systolic dysfunction in patients with CKD, exclusion of heart failure becomes important in this population. In the present study half of the patients had SHF along with impaired kidney and has significant implications for use of natriuretic peptide assay. Both plasma BNP and NT-proBNP levels correlated with NYHA symptom severity in patients with impaired kidney function. It has been consistently found in a large number of studies that BNP and NT-proBNP are elevated in patients with heart failure, and values were found to be related to disease severity as assessed by NYHA functional class [39,40]. Strong association was found between NT-proBNP levels and left ventricular hypertrophy and dysfunction in a recently reported large CKD cohort without heart failure. In the same study NT-proBNP improved the ability of clinical models to predict systolic dysfunction in CKD patients [41].

Overtly symptomatic patients (n = 28) in our study classified under NYHA class four had extremely high BNP and NT-proBNP levels (median 409.4 and 22520 pg/ml respectively). The biological activity of BNP includes diuresis, natriuresis, and inhibition of renin-angiotensin system, endothelin secretion, and systemic and renal sympathetic activity. Paradoxically, in congestive heart failure high BNP levels measured by conventional assays are associated with an absence of effect of this hormone [42]. The phenomena of ‘endocrine paradox’ of the heart are characterized by extremely high circulating levels of natriuretic peptides in heart failure patients, showing signs of fluid retention and vasoconstriction [43,44]. Some recent findings suggest that the commercially available immunoassay methods tend to progressively overestimate the real biological activity of the natriuretic peptides in patients with heart failure [45].

The studied population already suffered from impaired kidney function; hence duration of the disease may be a confounder in the current study. Ideally occult myocardial ischemia which raises BNP and NT-proBNP levels should have been excluded. Use of Cockcroft Gault equation to estimate GFR is controversial in Asians populations but good agreement between Cockcroft Gault and creatinine clearance has been observed in our setup [33]. Patients with CKD suffer from both systolic and diastolic heart failure but then in the current study patients with heart failure with preserved systolic function were not considered. Another limitation of the study is small sample size in subcategories of the data and further studies are required to reliably derive cutoffs. Differences exist in analytical performances of different BNP and MT-proBNP assays therefore it may be not right to suggest identical cut-off or decision values for all BNP/NT-proBNP immunoassays [46].

## Conclusion

Product inserts by manufacturers of plasma BNP and NT-proBNP only have a single cut-off used in normal population and there is no mention of limitations of using them in patients with compromised renal function. The fact remains that many patients with heart failure also have renal insufficiency. Therefore, current study becomes very important in making clinicians aware of how to interpret the results in concomitant renal disease and also for kit manufacturers to include it in their product labeling. Both natriuretic peptides’ assays can be used in patients with impaired kidney function but by using higher cutoffs stratified according to kidney dysfunction. NT-proBNP appears to predict SHF better than BNP. Kidney dysfunction makes the interpretation of these natriuretic peptides complicated. To improve the quality of care and subsequent prognosis, plasma BNP and especially NT-proBNP values should be interpreted in relation to the severity of kidney dysfunction.

### Ethical approval

The study was given approval by the Ethical Review Committee of the Aga Khan University Hospital. Reference number: 1054-Path-ERC-08).

## Competing interests

The authors declare that they have no competing interests.

## Authors’ contributions

LJ was involved in planning, patient recruitment, biochemical analysis, data analysis and write up of the manuscript, WK participated in patient selection and recruitment and review of manuscript, JT contributed in patient selection and recruitment and review of manuscript, IS was involved in project execution, data analysis and manuscript review, IA participated in design of study and statistical analysis of data, HS was involved in patient recruitment and FG proposed the initial idea, participated in its design, project execution and manuscript review. All authors read and approved the final manuscript.

## Pre-publication history

The pre-publication history for this paper can be accessed here:

http://www.biomedcentral.com/1471-2369/14/117/prepub
